# Supplementary material for: Media matters: phenol red and fetal bovine serum estrogen in traditional cell culture media influence human mesenchymal stromal cell (hMSC) processes and differentiation in a sex-biased manner
Source: Biol Sex Differ. 2026 May 19;17:132. doi: 10.1186/s13293-026-00921-w (PMC13366848; doi:10.1186/s13293-026-00921-w)
Supplement: Supplementary file 1 — Supplementary Material 1 [file 13293_2026_921_MOESM1_ESM.pdf]

CERTIFICATE OF ANALYSIS
FETAL BOVINE SERUM
Premium

Lot No: H19101

Catalog No: S 11195 (50 ml size)
S 11110 (100 ml size)
S 11150 (500 ml size)

triple 0.1μm filter processed serum

Expiration Date: August 2024 **Store Frozen at:** -5°C to -20°C

Notice: We collect and process our serum rapidly at cold temperatures to yield the highest quality serum with excellent cell growth properties. This cold processing leaves some fibrinogen in the serum which may convert to fibrin upon storage, thawing, or heat-inactivation. Fibrin can cause the serum to look slightly turbid or may be visible as a flocculent material. This material does not adversely affect the growth performance characteristics of the serum.

TEST	SPECIFICATION	RESULT
Bacterial and Fungal Testing (U.S.P. Atlanta Biologicals Modified)	*Not Detected*	Not Detected
Mycoplasma Testing (Large Volume Barile Method)	*Not Detected*	Not Detected
Mycoplasma Testing, Supplemental (DNA Fluorochrome Stain)	*Not Detected*	Not Detected
Virus Testing (Modified 9CFR)		
BVDV - Fluorescent Antibody	*Test*	Tested
Cytopathogenic Agents- e.g. IBRV	*Test And Report*	Not Detected
Hemadsorbing Agents - e.g. PI-3V	*Test And Report*	Not Detected
BTV	*Not Detected*	Not Detected
Cell Culture Testing	*Pass*	Pass
pH	*6.8 - 7.8*	7.4
Osmolality (Vapor Pressure)	*280 - 335 mOsm*	307.7 mOsm
Endotoxin (Limulus Amebocyte Lysate Gel Clot)	*≤50.0 EU/ml*	< 0.06 EU/ml
Hemoglobin (Spectrophotometric)	*≤25 mg/dl*	19.1 mg/dl

This product is manufactured for research and development purposes only. It is not intended for any human or animal diagnostic, therapeutic or other clinical uses. It is also not for agricultural, food, drug, cosmetic or household use. The use of these products must be supervised by a person technically qualified to handle potentially hazardous material.

Atlanta Biologicals™ Serum and Cell Culture Products are now part of R&D Systems, a Bio-Techne Brand.

For research use or manufacturing purposes only. Trademarks and registered trademarks are the property of their respective owners.

FETAL BOVINE SERUM

Premium

Lot No: H19101

Catalog No: S 11195 (50 ml size)
S 11110 (100 ml size)
S 11150 (500 ml size)

BIOCHEMICAL PROFILE	SPECIFICATION	RESULT
Total Protein	*3.0 to 4.6 g/dl*	3.5 g/dl
Albumin	*check and record*	2.3 g/dl
Globulin	*check and record*	1.2 g/dl
A/G ratio	*check and record*	1.9
IgG	*check and record*	9.0 mg/dl
ALT/SGPT	*check and record*	9.0 IU/l
GGT	*check and record*	3.0 IU/l
Alkaline Phosphatase	*check and record*	279.0 IU/l
Total Bilirubin	*check and record*	0.1 mg/dl
Iron	*check and record*	180.0 µg/dl
UIBC	*check and record*	73.0 µg/dl
Cholesterol	*check and record*	39.0 mg/dl
Triglycerides	*check and record*	72.0 mg/dl
Glucose	*check and record*	59.0 mg/dl
Blood Urea Nitrogen (BUN)	*check and record*	21.0 mg/dl
Creatinine	*check and record*	2.7 mg/dl
BUN/Creatinine Ratio	*check and record*	8.0
Uric Acid	*check and record*	0.9 mg/dl
Sodium	*check and record*	138.0 meq/l
Potassium	*check and record*	11.6 meq/l
Sodium/Potassium Ratio	*check and record*	11.9
Chloride	*check and record*	101.0 meq/l
Calcium	*check and record*	13.9 mg/dl
Phosphorus	*check and record*	10.2 mg/dl
Magnesium	*check and record*	3.6 mg/dl
Bicarbonate	*check and record*	15.0 mmol/l

This product is manufactured for research and development purposes only. It is not intended for any human or animal diagnostic, therapeutic or other clinical uses. It is also not for agricultural, food, drug, cosmetic or household use. The use of these products must be supervised by a person technically qualified to handle potentially hazardous material.

Atlanta Biologicals™ Serum and Cell Culture Products are now part of R&D Systems, a Bio-Techne Brand.

For research use or manufacturing purposes only. Trademarks and registered trademarks are the property of their respective owners.

FETAL BOVINE SERUM
Premium

Lot No: H19101

Catalog No: S 11195 (50 ml size)
S 11110 (100 ml size)
S 11150 (500 ml size)

The Fetal Bovine Serum used in Atlanta Biologicals' manufacturing process is certified as meeting all U.S.D.A. requirements for donor animal health, country of origin, and traceability of the product.

Origin: The Fetal Bovine Serum is collected in U.S.D.A. approved slaughterhouses or in countries certified by the U.S.D.A. to be free of Foot and Mouth Disease (FMD), Bovine Spongiform Encephalopathy (BSE) and other exotic disease agents. All imported serum used for manufacturing purposes is tested and approved by the U.S.D.A. for distribution in the United States.

ORIGIN: Central America

Donor Animals: All fetal blood is collected from fetuses derived from healthy animals. The donor dams must pass both ante- and post-mortem certified veterinary inspections before collection of the fetal blood.

Traceability: The serum has been imported into the United States in full compliance with all USDA import regulations. Final processing of the serum took place in Atlanta Biologicals' processing facility located in the United States. All Fetal Bovine Serum is traceable back to the date and location of collection.

Signature On File	September 16, 2019
Quality Control Department	Date

This product is manufactured for research and development purposes only. It is not intended for any human or animal diagnostic, therapeutic or other clinical uses. It is also not for agricultural, food, drug, cosmetic or household use. The use of these products must be supervised by a person technically qualified to handle potentially hazardous material.

Atlanta Biologicals™ Serum and Cell Culture Products are now part of R&D Systems, a Bio-Techne Brand.

For research use or manufacturing purposes only. Trademarks and registered trademarks are the property of their respective owners.
