# Supplementary material for: Media matters: phenol red and fetal bovine serum estrogen in traditional cell culture media influence human mesenchymal stromal cell (hMSC) processes and differentiation in a sex-biased manner
Source: Biol Sex Differ. 2026 May 19;17:132. doi: 10.1186/s13293-026-00921-w (PMC13366848; doi:10.1186/s13293-026-00921-w)
Supplement: Supplementary file 2 — Supplementary Material 2 [file 13293_2026_921_MOESM2_ESM.pdf]

CERTIFICATE OF ANALYSIS
FETAL BOVINE SERUM
Charcoal Dextran Treated

Catalog No: S 11695 (50 ml size)
S 11610 (100 ml size)
S 11650 (500 ml size)

Lot No: J18126

0.1 μm filter processed serum

Expiration Date: September 2023 **Store Frozen at:** -5°C to -20°C

TEST	SPECIFICATION	RESULT
Bacterial and Fungal Testing (USP 71)	*NOT DETECTED*	NOT DETECTED
Mycoplasma Testing	*NOT DETECTED*	NOT DETECTED
Virus Testing		
BVDV	*NOT DETECTED*	NOT DETECTED
Cytopathogenic Agents- e.g. IBRV	*NOT DETECTED*	NOT DETECTED
Hemadsorbing Agents - e.g. PI-3V	*NOT DETECTED*	NOT DETECTED
CPE	*NOT DETECTED*	NOT DETECTED
pH	*6.5 - 8.5*	7.5
Osmolality	*260 - 350 mOsm*	304.0 mOsm
Endotoxin	*≤ 50.0 EU/ml*	< 0.10 EU/ml
Hemoglobin	*≤ 25 mg/dl*	7.0 mg/dl

This product is manufactured for research and development purposes only. It is not intended for any human or animal diagnostic, therapeutic or other clinical uses. It is also not for agricultural, food, drug, cosmetic or household use. The use of these products must be supervised by a person technically qualified to handle potentially hazardous material.

Atlanta Biologicals™ Serum and Cell Culture Products are now part of R&D Systems, a Bio-Techne Brand.

For research use or manufacturing purposes only. Trademarks and registered trademarks are the property of their respective owners.

FETAL BOVINE SERUM

Charcoal Dextran Treated

Lot No: J18126

Catalog No: S 11695 (50 ml size)
S 11610 (100 ml size)
S 11650 (500 ml size)

Hormone Profile	*Specification*	Pre-Treatment Value	Post-Treatment Value
Testosterone	*check and record*	< 0.01 ng/ml	< 0.01 ng/ml
Estradiol	*check and record*	20.3 pg/ml	< 15.0 pg/ml
Insulin	*check and record*	6.0 μIU/ml	< 2.7 μIU/ml
Cortisol	*check and record*	< 0.20 μg/dl	< 0.20 μg/dl
Progesterone	*check and record*	< 0.20 ng/ml	< 0.20 ng/ml
T3	*check and record*	95.9 ng/dl	< 10.0 ng/dl
T4	*check and record*	16.0 μg/dl	5.0 μg/dl

Biochemical Profile	*Specification*	Result
Total Protein	*3.2 to 4.6 g/dl*	3.6 g/dl
Albumin	*check and record*	2.1 g/dl
Globulin	*check and record*	1.5 g/dl
GGT	*check and record*	5.0 IU/l
AST (SGOT)	*check and record*	43.0 IU/l
ALT (SGPT)	*check and record*	5.0 IU/l
Alkaline Phosphatase	*check and record*	287.0 IU/l
Total Bilirubin	*check and record*	0.1 mg/dl
Iron	*check and record*	172.0 μg/dl
Cholesterol	*check and record*	30.0 mg/dl
Triglycerides	*check and record*	63.0 mg/dl
Glucose	*check and record*	50.0 mg/dl
Blood Urea Nitrogen (BUN)	*check and record*	17.0 mg/dl
Creatinine	*check and record*	0.0 mg/dl
Uric Acid	*check and record*	0.0 mg/dl
Sodium	*check and record*	139.0 meq/l
Potassium	*check and record*	> 10.0 meq/l
Chloride	*check and record*	101.0 meq/l
Calcium	*check and record*	11.8 mg/dl
Phosphorus	*check and record*	9.4 mg/dl
Magnesium	*check and record*	2.9 mg/dl

This product is manufactured for research and development purposes only. It is not intended for any human or animal diagnostic, therapeutic or other clinical uses. It is also not for agricultural, food, drug, cosmetic or household use. The use of these products must be supervised by a person technically qualified to handle potentially hazardous material.

Atlanta Biologicals™ Serum and Cell Culture Products are now part of R&D Systems, a Bio-Techne Brand.

For research use or manufacturing purposes only. Trademarks and registered trademarks are the property of their respective owners.

ATLANTA biologicals by R&D SYSTEMS

For inquiries visit: atlantabio.com
Or call: (800) 780-7788 (770) 446-3336

FETAL BOVINE SERUM

Charcoal Dextran Treated

Lot No: J18126

**Catalog No:**	S 11695	(50 ml size)
	S 11610	(100 ml size)
	S 11650	(500 ml size)

The Fetal Bovine Serum used in manufacturing the product stated above is certified by the manufacturer as meeting all of the U.S.D.A requirements for donor animal health, country of origin, and traceability of the product.

Origin: The fetal bovine serum used in the production of this lot was collected in U.S.D.A. inspected slaughterhouses in the USA.

Donor Animals: All fetal blood is collected from fetuses derived from healthy animals. The donor dams must pass both ante- and post-mortem certified veterinary inspections before collection of the fetal blood.

Traceability: All of the Fetal Bovine Serum is traceable back to the date and location of collection.

Signature On File	*November 4, 2018*
Quality Control Department	Date

This product is manufactured for research and development purposes only. It is not intended for any human or animal diagnostic, therapeutic or other clinical uses. It is also not for agricultural, food, drug, cosmetic or household use. The use of these products must be supervised by a person technically qualified to handle potentially hazardous material.

Atlanta Biologicals™ Serum and Cell Culture Products are now part of R&D Systems, a Bio-Techne Brand.

For research use or manufacturing purposes only. Trademarks and registered trademarks are the property of their respective owners.
