# Supplementary material for: Media matters: phenol red and fetal bovine serum estrogen in traditional cell culture media influence human mesenchymal stromal cell (hMSC) processes and differentiation in a sex-biased manner
Source: Biol Sex Differ. 2026 May 19;17:132. doi: 10.1186/s13293-026-00921-w (PMC13366848; doi:10.1186/s13293-026-00921-w)

Supplementary Figure 1: Proliferation


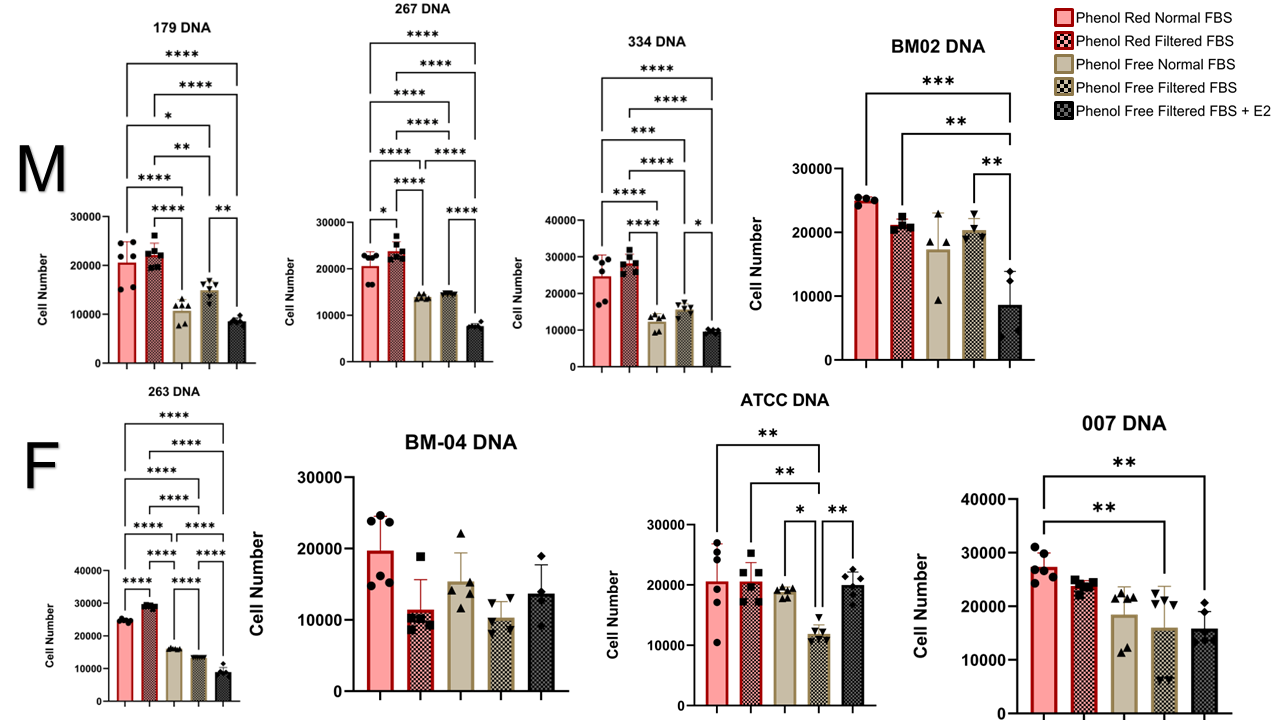
**Figure 1**: Individual Graphs for Proliferation assayed VIA Picogreen Statistical comparisons done within individual donors. All data tested for normality utilizing the skewness test (s < .5) and analyzed utilizing 2-way ANOVA, and Bonferroni multiple comparison testing. ANOVA Statistics * P =< .05, ** P =< .01, *** P =< .001, **** P =< .0001. Each dot represents a different well in a tissue culture plate, 3 wells per donor, 4 donors, for a total of 12 points per condition.

Supplementary Figure 2: Metabolism by Prestoblue


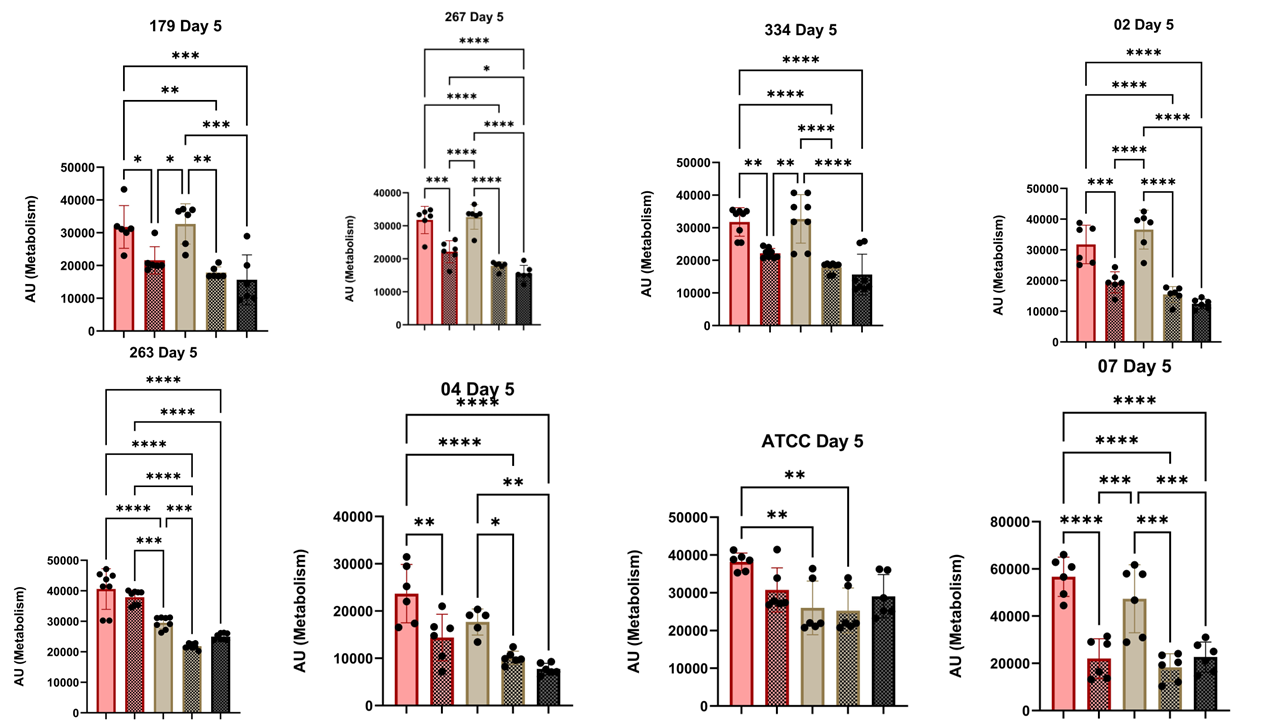
**Figure 2**: Individual Graphs for Metabolism assayed VIA Prestoblue Statistical comparisons done within individual donors. All data tested for normality utilizing the skewness test (s < .5) and analyzed utilizing 2-way ANOVA, and Bonferroni multiple comparison testing. ANOVA Statistics * P =< .05, ** P =< .01, *** P =< .001, **** P =< .0001. Each dot represents a different well in a tissue culture plate, 6-8 wells per donor, 4 donors per sex.

Supplementary Figure 3: Seahorse Basal Metabolic Rate


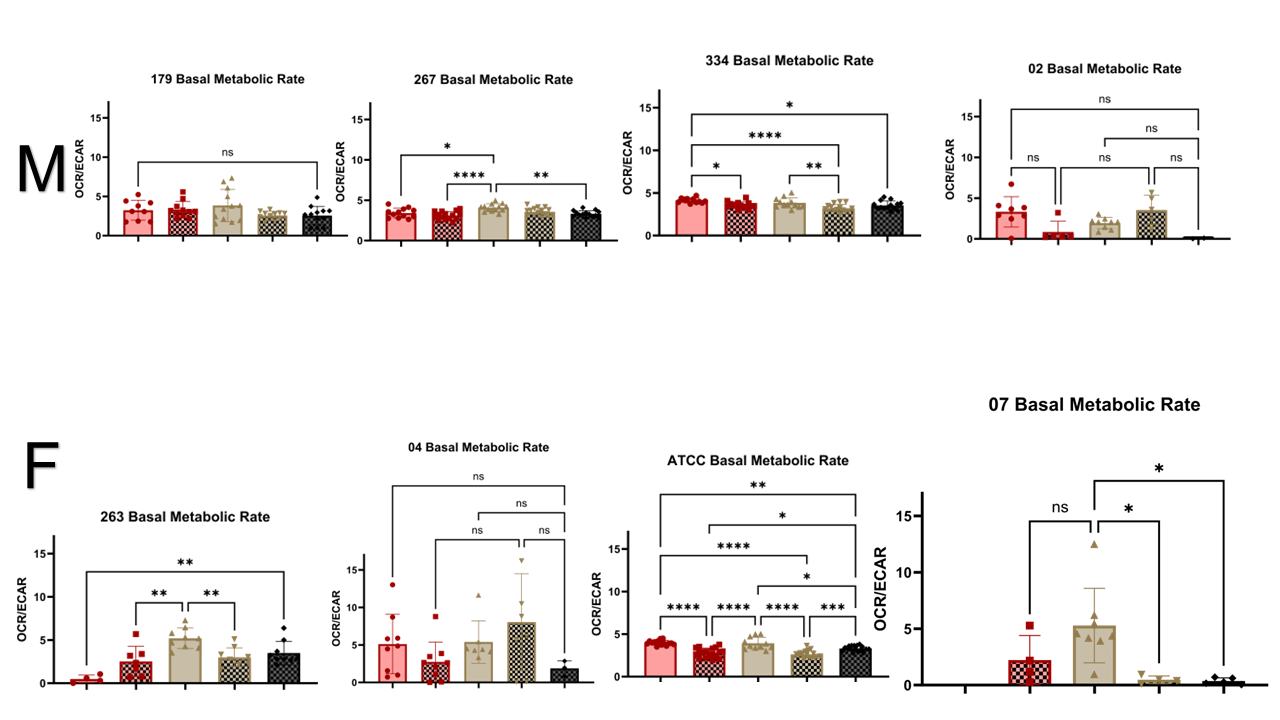
**Figure 3**: Individual Graphs for Metabolism assayed VIA Seahorse Basal Metabolic Rate Statistical comparisons done within individual donors. All data tested for normality utilizing the skewness test (s < .5) and analyzed utilizing 2-way ANOVA, and Bonferroni multiple comparison testing. ANOVA Statistics * P =< .05, ** P =< .01, *** P =< .001, **** P =< .0001. Each dot represents a different well in a tissue culture plate, 6-12 wells per donor, 4 donors per sex.

Supplementary Figure 4: Senescence Associated Staining


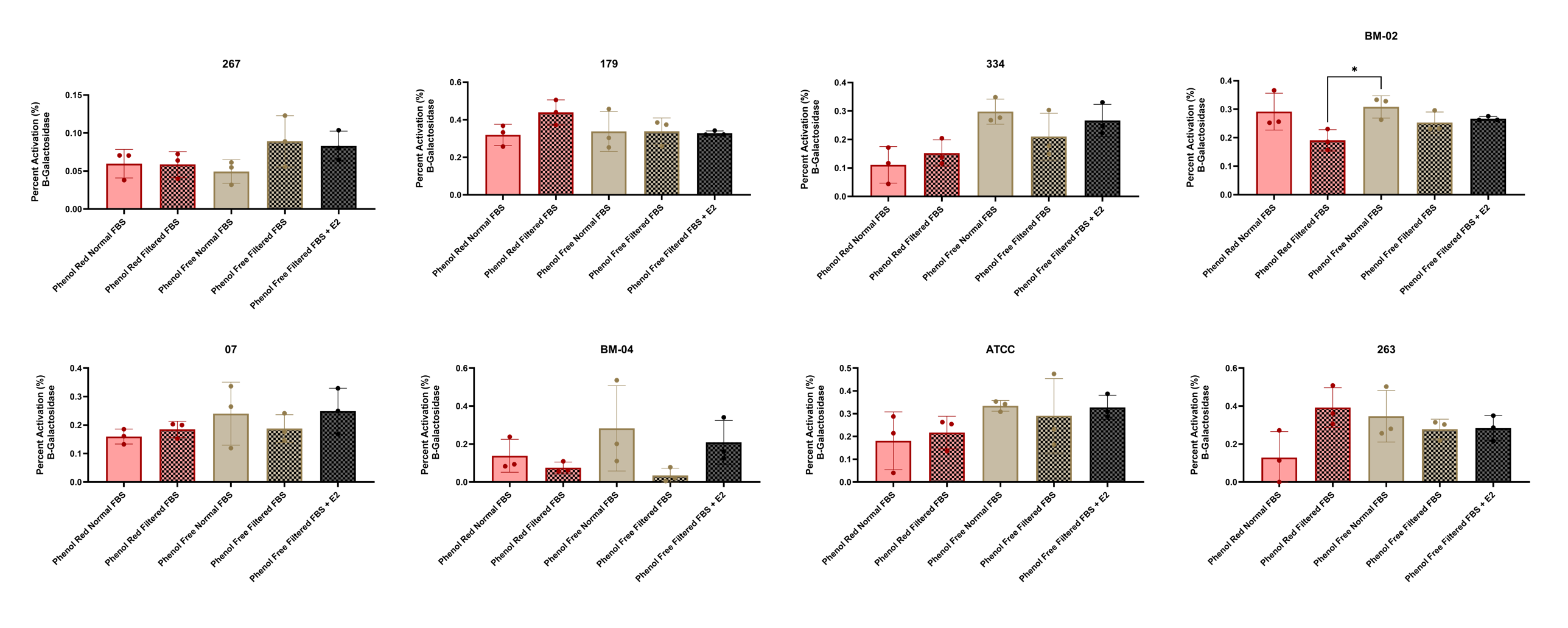


**Figure 4**: Individual Graphs for Senesence assayed β-Galactosidase Staining. Statistical comparisons done within individual donors. All data tested for normality utilizing the skewness test (s < .5) and analyzed utilizing 2-way ANOVA, and Bonferroni multiple comparison testing. ANOVA Statistics * P =< .05, ** P =< .01, *** P =< .001, **** P =< .0001. Each dot represents a different well in a tissue culture plate, 3 wells per donor, 4 donors per sex.

Supplementary Figure 5: Alizarin Red Staining


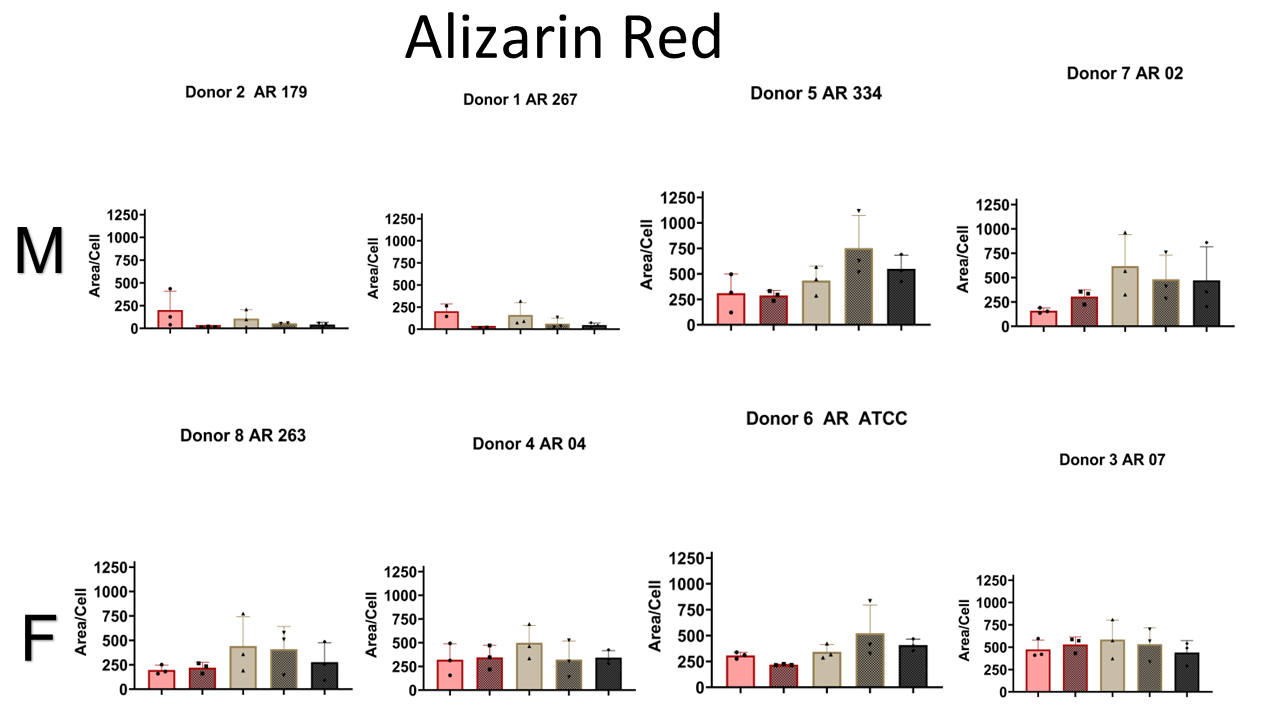
**Figure 5**: Individual Graphs for Osteogenic Differentiation assayed Alizarin Red Staining. Statistical comparisons done within individual donors. All data tested for normality utilizing the skewness test (s < .5) and analyzed utilizing 2-way ANOVA, and Bonferroni multiple comparison testing. ANOVA Statistics * P =< .05, ** P =< .01, *** P =< .001, **** P =< .0001. Each dot represents a different well.

Supplementary Figure 6: Oil Red O Staining


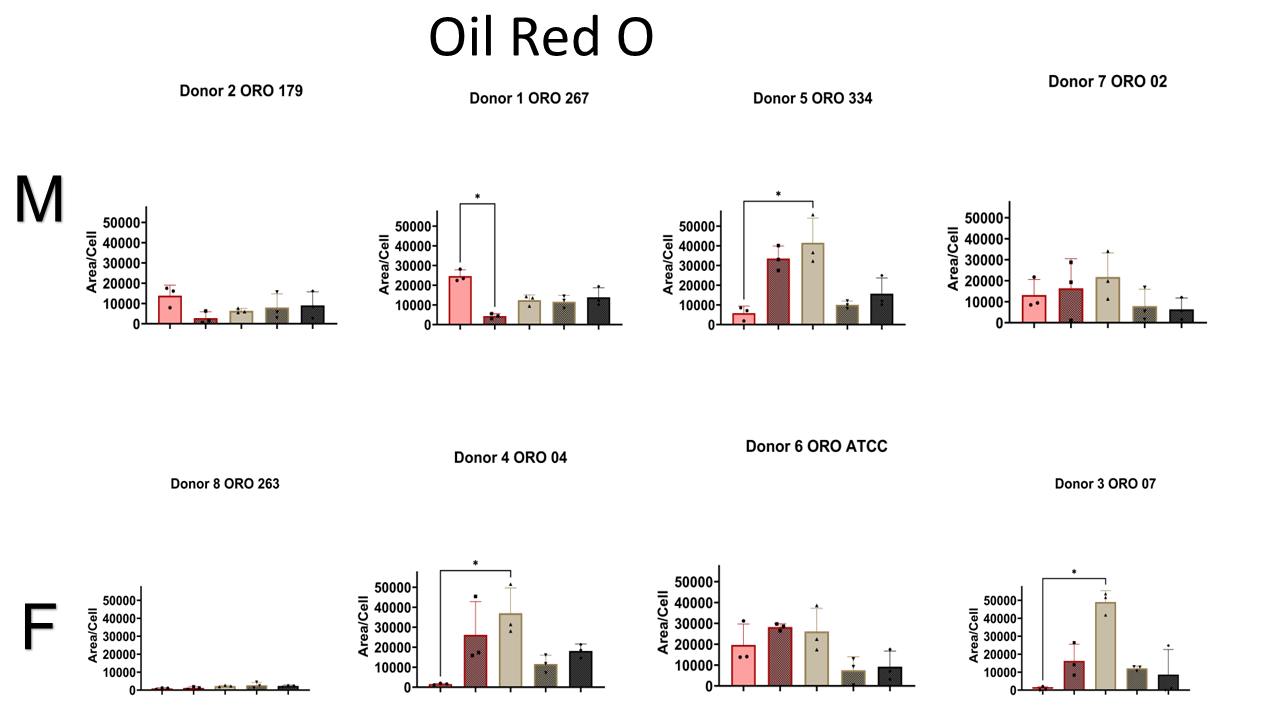
**Figure 6**: Individual Graphs for Adipogenic Differentiation assayed Oil Red O Staining. Statistical comparisons done within individual donors. All data tested for normality utilizing the skewness test (s < .5) and analyzed utilizing 2-way ANOVA, and Bonferroni multiple comparison testing. ANOVA Statistics * P =< .05, ** P =< .01, *** P =< .001, **** P =< .0001. Each dot represents a different well comprising approximately 25 images.

Supplementary Figure 7: ESR1 qPCR


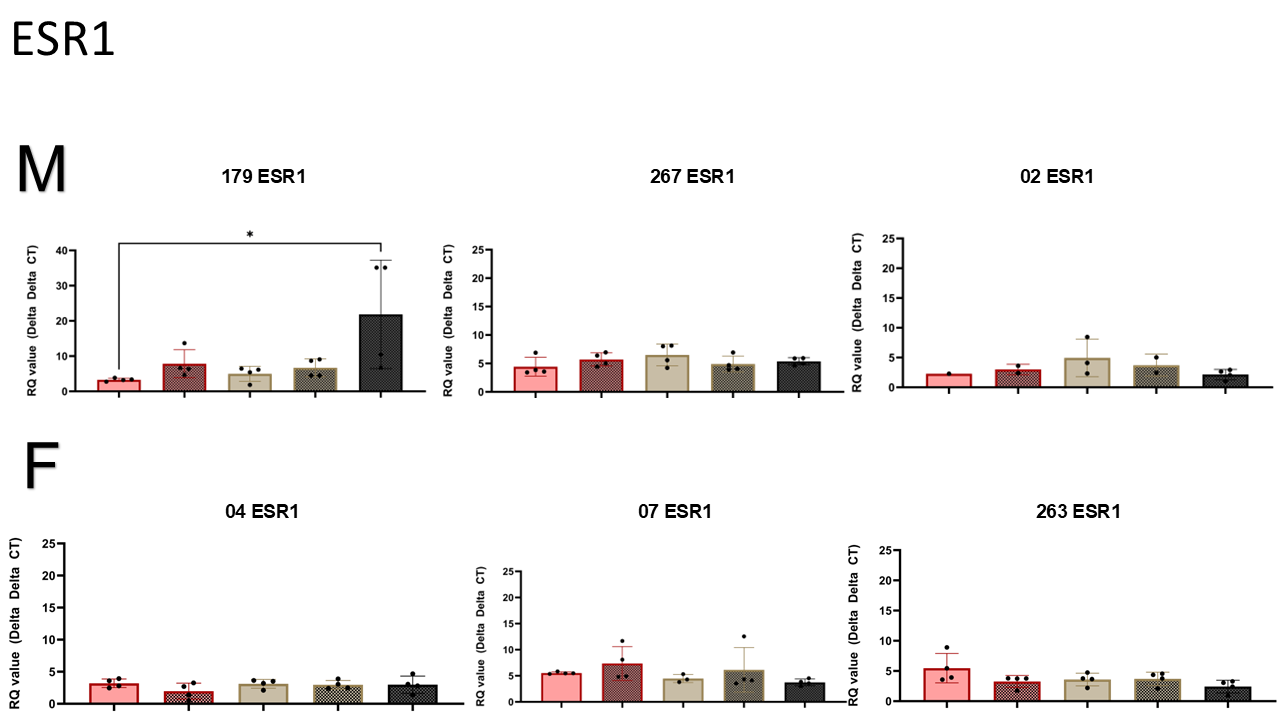
**Figure 7**: Individual Graphs for ESR1 expression assayed qPCR. Statistical comparisons done within individual donors. All data tested for normality utilizing the skewness test (s < .5) and analyzed utilizing 2-way ANOVA, and Bonferroni multiple comparison testing. ANOVA Statistics * P =< .05, ** P =< .01, *** P =< .001, **** P =< .0001. Each dot represents a different image from a well in a tissue culture plate, 4 wells per donor, 4 donors per sex.

Supplementary Figure 8: COL1a1 qPCR


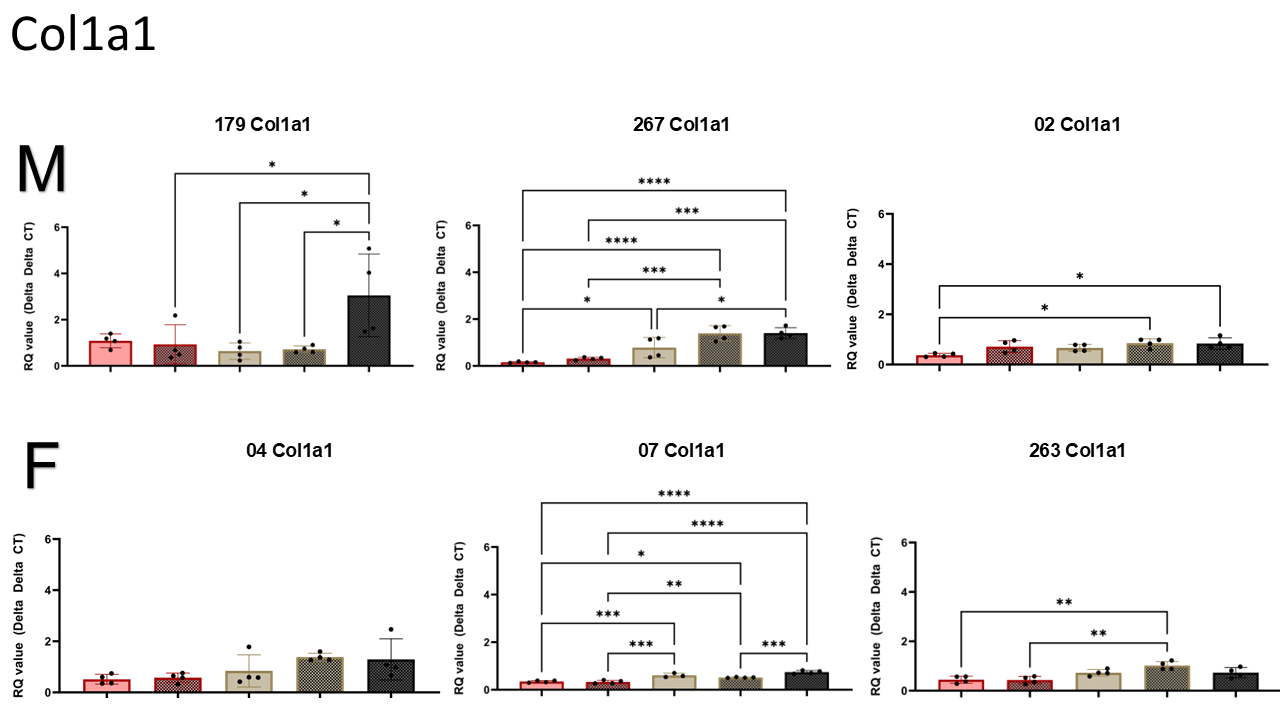
**Figure 8**: Individual Graphs for COL1a1 expression assayed qPCR. Statistical comparisons done within individual donors. All data tested for normality utilizing the skewness test (s < .5) and analyzed utilizing 2-way ANOVA, and Bonferroni multiple comparison testing. ANOVA Statistics * P =< .05, ** P =< .01, *** P =< .001, **** P =< .0001. Each dot represents a different image from a well in a tissue culture plate, 4 wells per donor, 4 donors per sex.

Supplementary Figure 9: RUNX2 qPCR

**
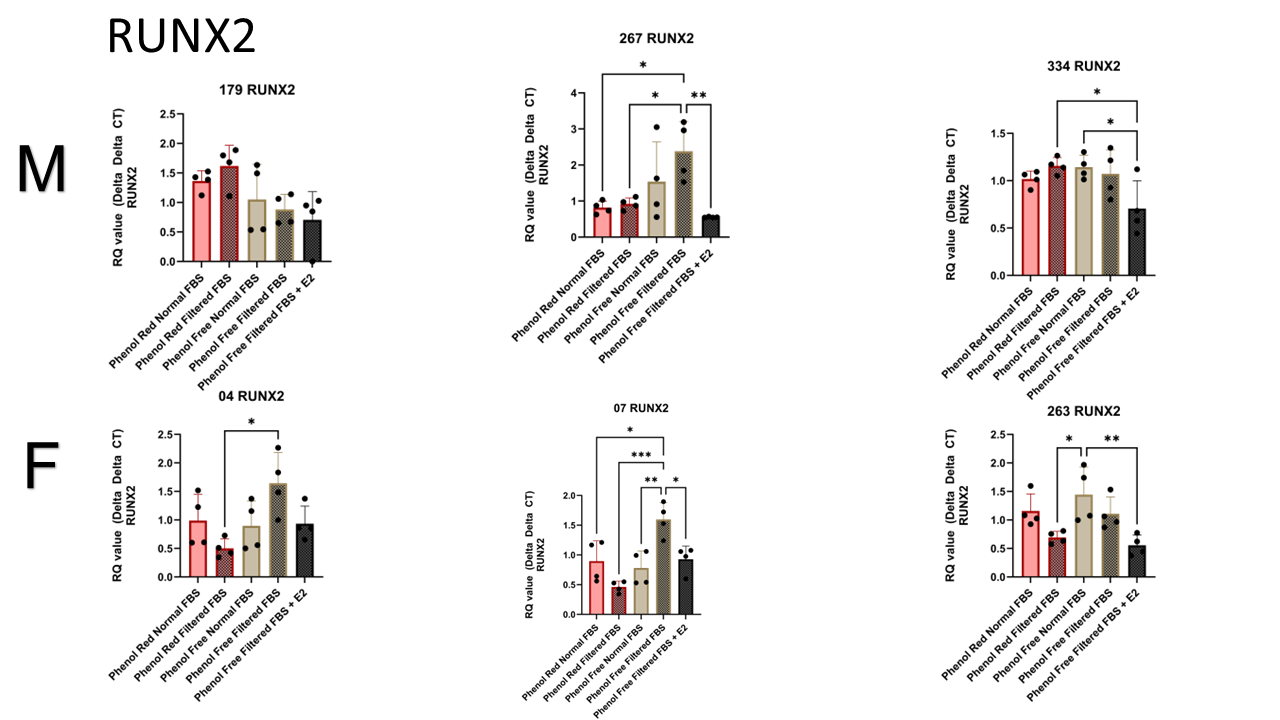
Figure 9**: Individual Graphs for RUNX2 expression assayed qPCR. Statistical comparisons done within individual donors. All data tested for normality utilizing the skewness test (s < .5) and analyzed utilizing 2-way ANOVA, and Bonferroni multiple comparison testing. ANOVA Statistics * P =< .05, ** P =< .01, *** P =< .001, **** P =< .0001. Each dot represents a different image from a well in a tissue culture plate, 4 wells per donor, 4 donors per sex.

Supplementary Figure 10: COL10a1 qPCR

**
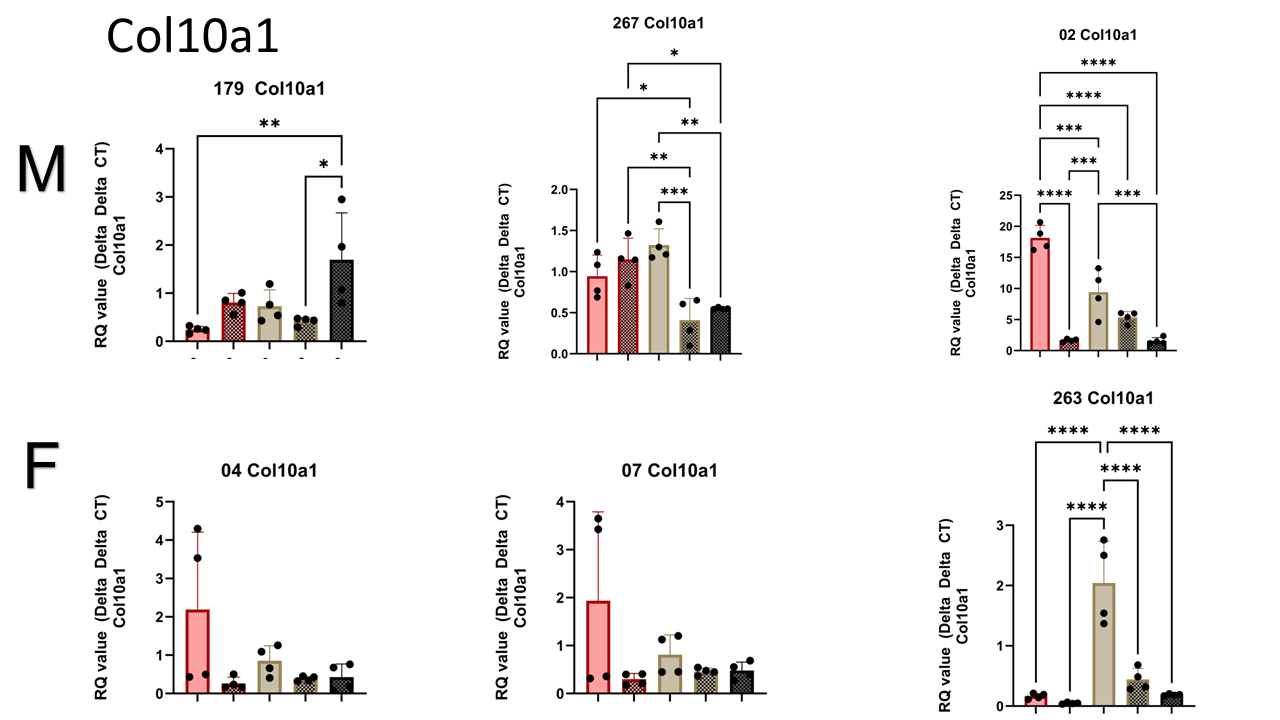
Figure 10**: Individual Graphs for COL10a1 expression assayed qPCR. Statistical comparisons done within individual donors. All data tested for normality utilizing the skewness test (s < .5) and analyzed utilizing 2-way ANOVA, and Bonferroni multiple comparison testing. ANOVA Statistics * P =< .05, ** P =< .01, *** P =< .001, **** P =< .0001. Each dot represents a different image from a well in a tissue culture plate, 4 wells per donor, 4 donors per sex.

Supplementary Figure 11: FABP4 qPCR

**
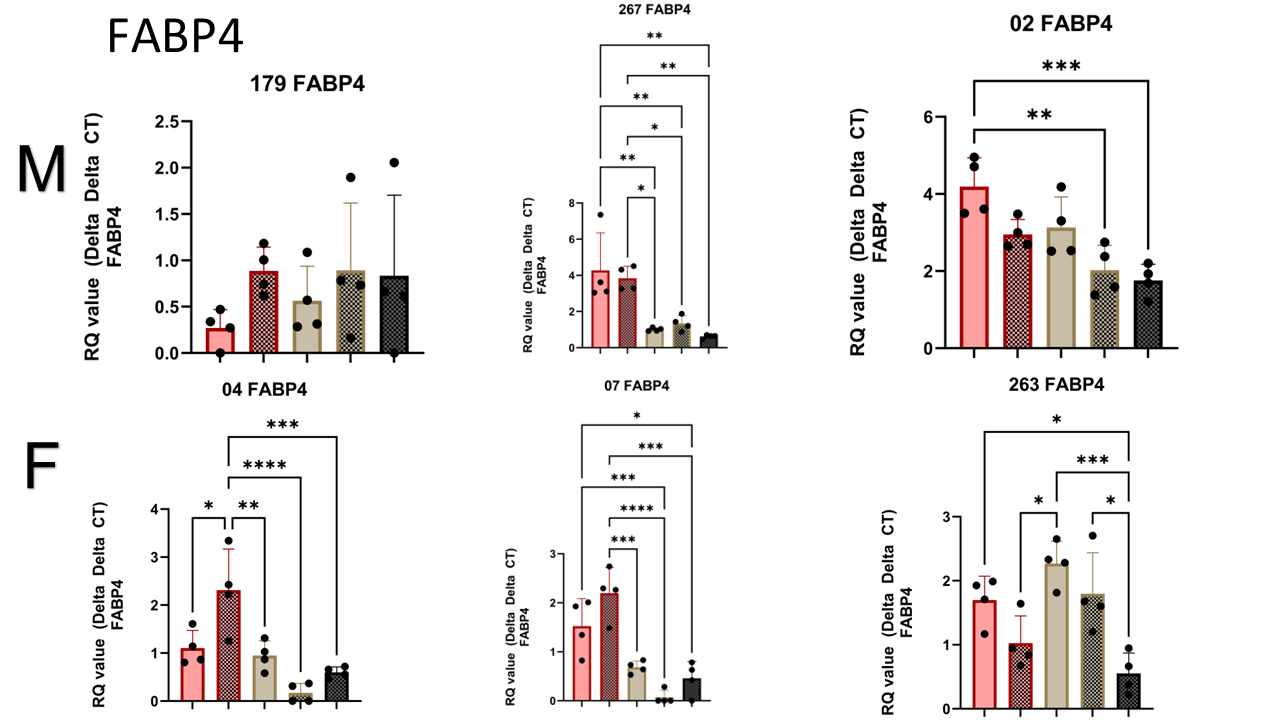
Figure 12**: Individual Graphs for FABP4 expression assayed qPCR. Statistical comparisons done within individual donors. All data tested for normality utilizing the skewness test (s < .5) and analyzed utilizing 2-way ANOVA, and Bonferroni multiple comparison testing. ANOVA Statistics * P =< .05, ** P =< .01, *** P =< .001, **** P =< .0001. Each dot represents a different image from a well in a tissue culture plate, 4 wells per donor, 4 donors per sex.

Supplementary Figure 12: PPARγ qPCR

**
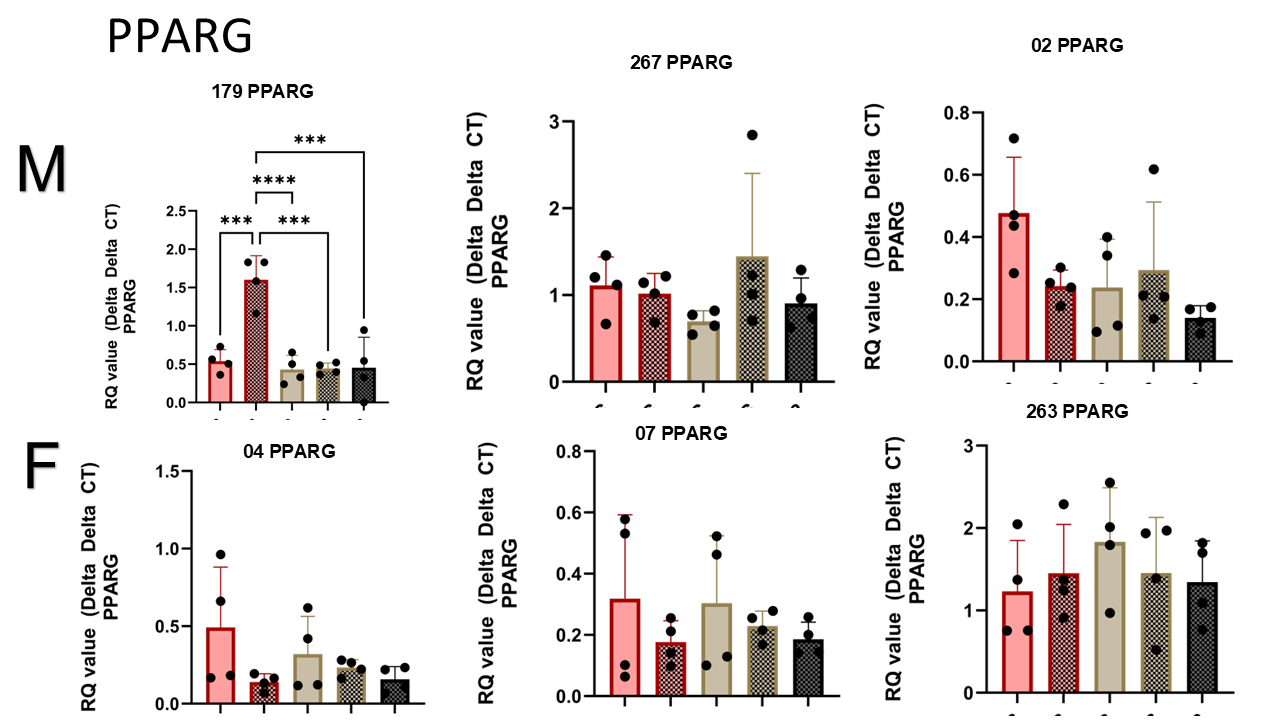
Figure 12**: Individual Graphs for PPARγ expression assayed qPCR. Statistical comparisons done within individual donors. All data tested for normality utilizing the skewness test (s < .5) and analyzed utilizing 2-way ANOVA, and Bonferroni multiple comparison testing. ANOVA Statistics * P =< .05, ** P =< .01, *** P =< .001, **** P =< .0001. Each dot represents a different image from a well in a tissue culture plate, 4 wells per donor, 4 donors per sex.

Supplementary Figure 13: SOX9 qPCR


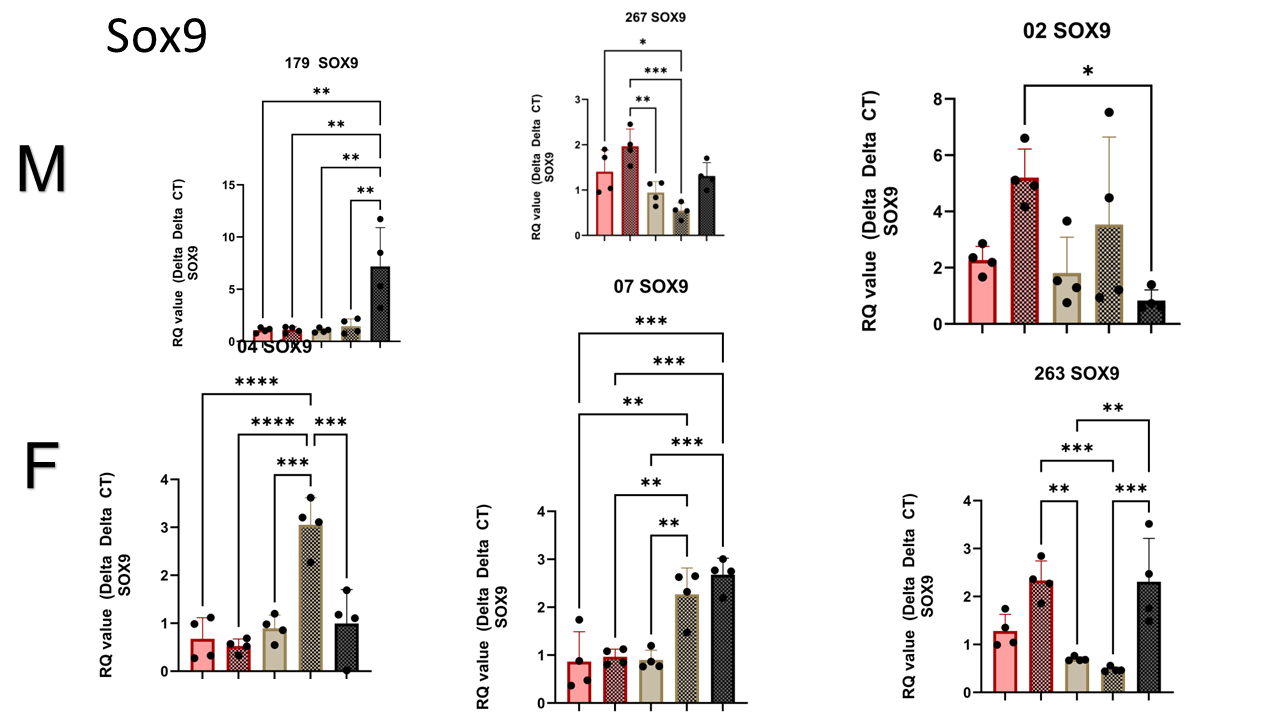
**Figure 13**: Individual Graphs for SOX9 expression assayed qPCR. Statistical comparisons done within individual donors. All data tested for normality utilizing the skewness test (s < .5) and analyzed utilizing 2-way ANOVA, and Bonferroni multiple comparison testing. ANOVA Statistics * P =< .05, ** P =< .01, *** P =< .001, **** P =< .0001. Each dot represents a different image from a well in a tissue culture plate, 4 wells per donor, 4 donors per sex.

**Supplementary Figure 14. DNA Data Individual Donors**


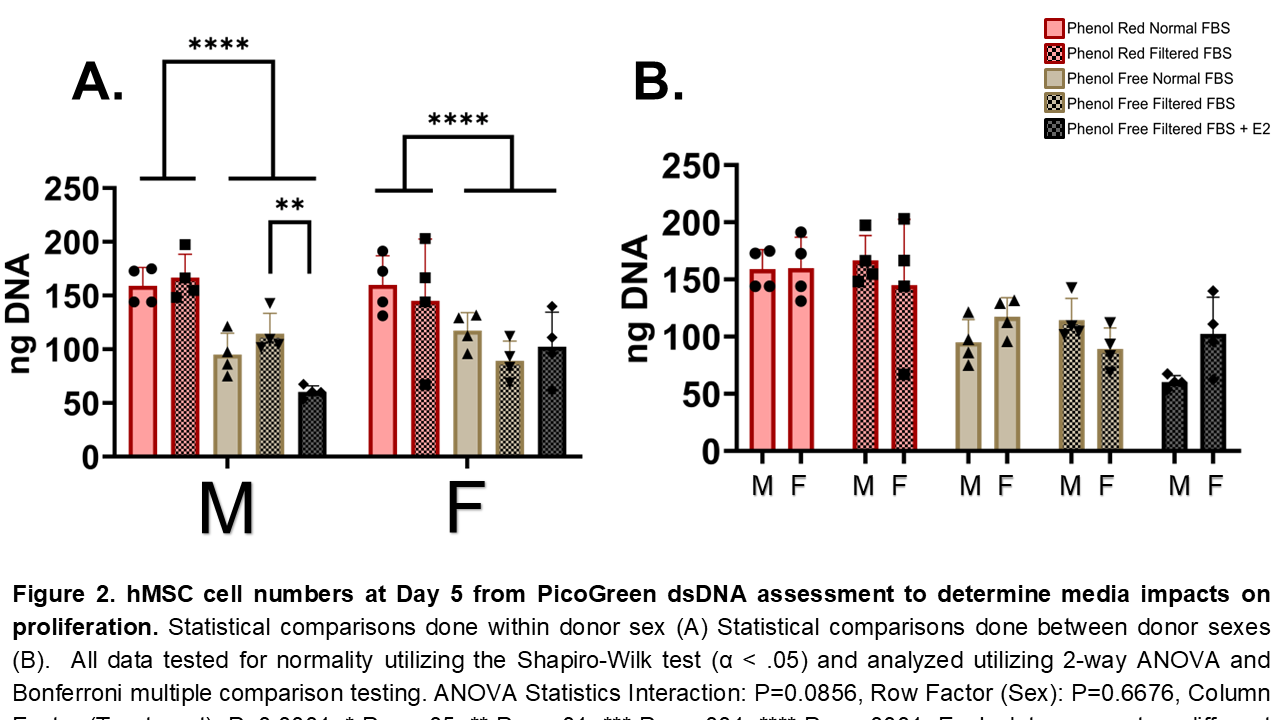
**Figure 14. hMSC cell numbers at Day 5 from PicoGreen dsDNA assessment to determine media impacts on proliferation.** Statistical comparisons done within donor sex (A) Statistical comparisons done between donor sexes (B). All data tested for normality utilizing the Shapiro-Wilk test (α = .05) and analyzed utilizing 2-way ANOVA and Bonferroni multiple comparison testing. See linear model for significance statistics P<0.0001. * P =< .05, ** P =< .01, *** P =< .001, **** P =< .0001. Each dot represents a different donor

**Supplementary Figure. 15 Prestoblue Individual Donors**


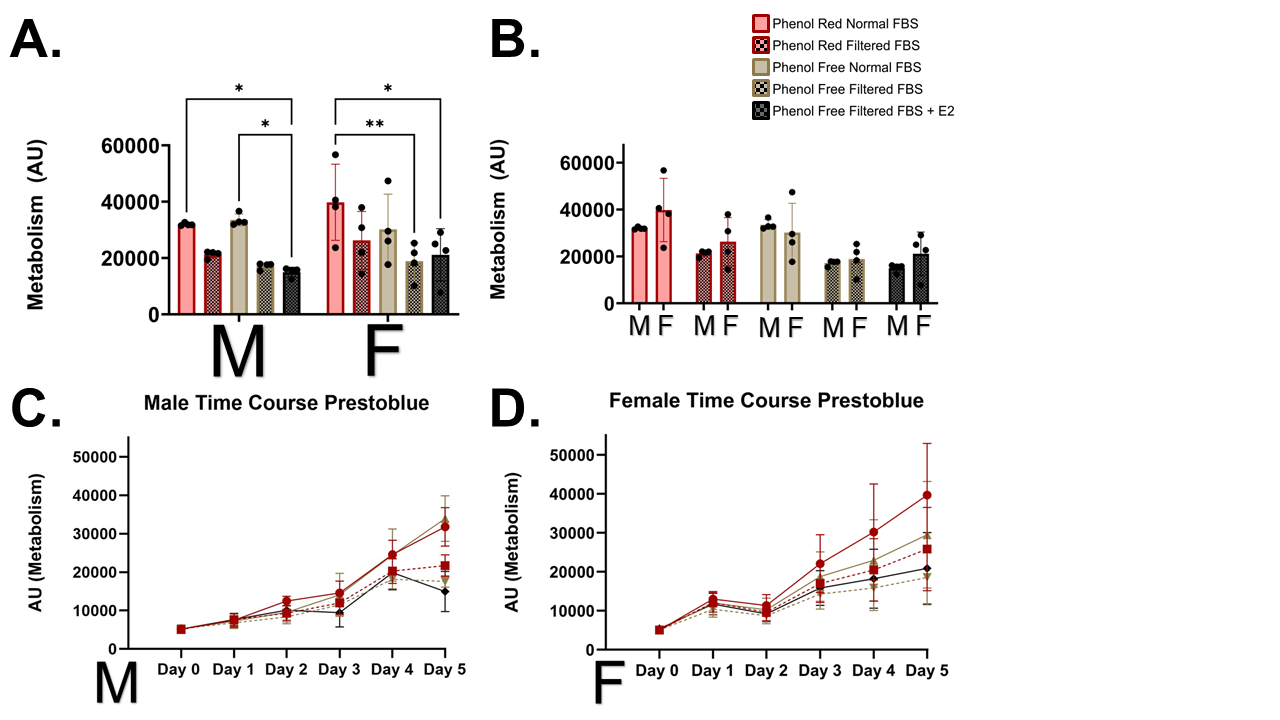
**Figure 15.** **hMSC PrestoBlue metabolism through Day 5.** Statistical comparisons done within donor sex (A). Statistical comparisons done between donor sexes (B). Male time course metabolism data through Day 5 (C). Female time course metabolism data through Day 5 (D). All data tested for normality utilizing the Shapiro-Wilk test (α = .05) and analyzed utilizing linear model analysis. See table for significance statistics. Each dot represents a different donor

**Supplementary Figure. 16 Seahorse Individual Donors**


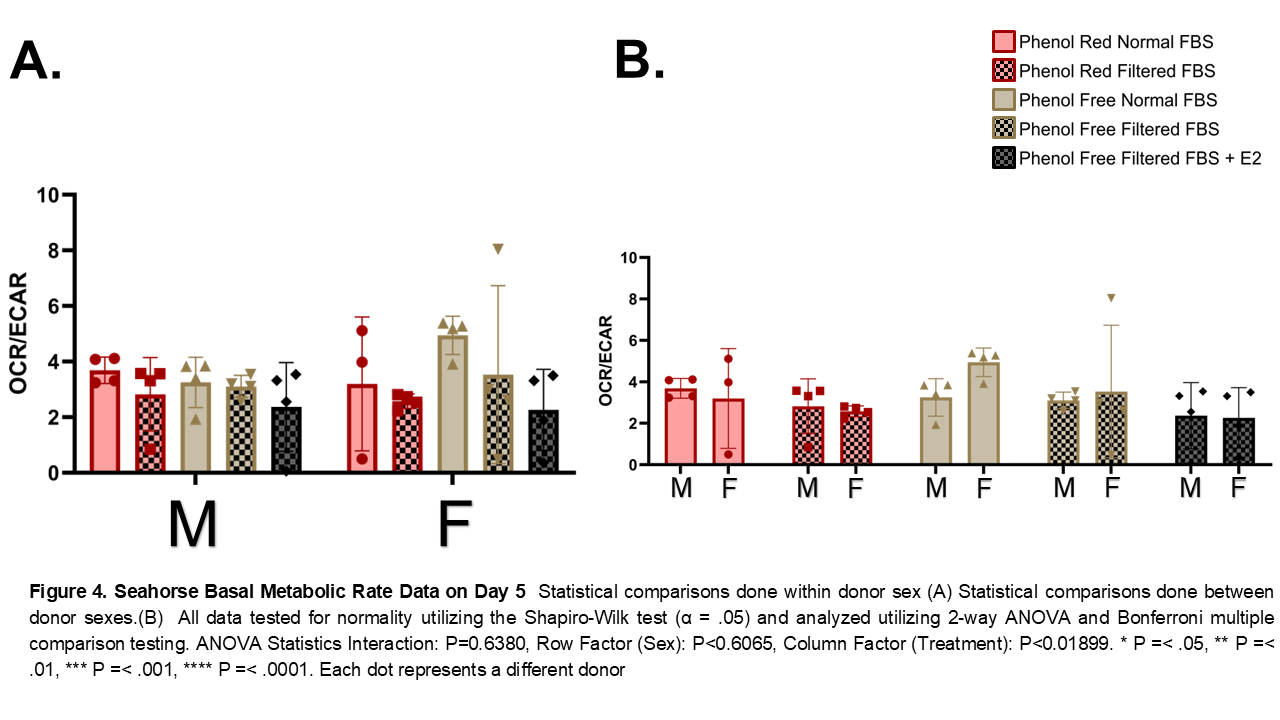
**Figure 16. Seahorse Basal Metabolic Rate Data on Day 5.** Statistical comparisons done within donor sex (A) Statistical comparisons done between donor sexes(B). All data tested for normality utilizing the Shapiro-Wilk test (α = .05) and analyzed utilizing linear model analysis. See table for linear model statistics. * P =< .05, ** P =< .01, *** P =< .001, **** P =< .0001. Each dot represents a different donor.


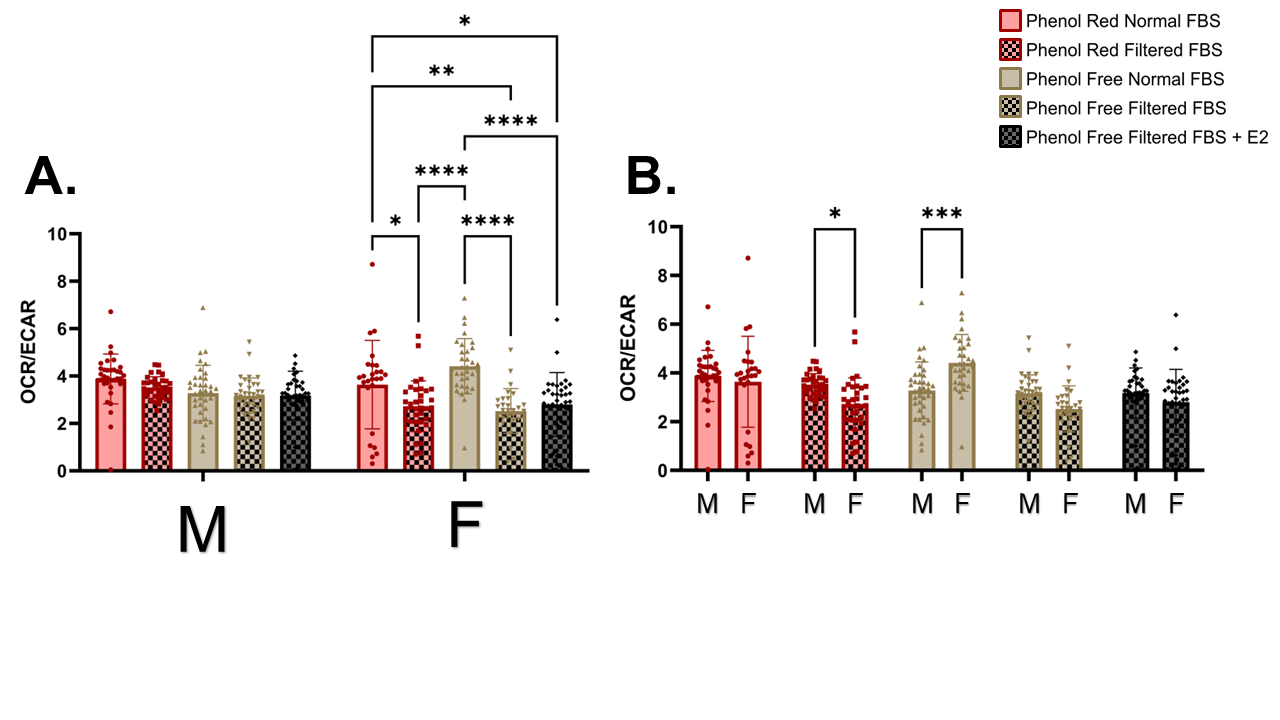


**Figure 16. Seahorse Basal Metabolic Rate Data on Day 5.** Statistical comparisons done within donor sex (A) Statistical comparisons done between donor sexes(B). All data tested for normality utilizing the Shapiro-Wilk test (α = .05) and analyzed utilizing linear model analysis. Seahorse Linear Model statistics Phenol: P=5.78e^-6^, FBS: P=5.10e^-17^, E2: P=6.60e^-6^, Sex: P=1.89e^-8^ Interactions: Phenol:FBS: P=0.849, Phenol:Sex: P=0.480, FBS:Sex: P=0.101, E2:Sex: P=0.407, Phenol:FBS:Sex: P=0.896* P =< .05, ** P =< .01, *** P =< .001, **** P =< .0001. Each dot represents a different well in a tissue culture plate, 6 wells per donor, 4 donors, for a total of 24 points per condition.

**Supplementary Figure. 17 Senescence Associated Staining Individual Donors**


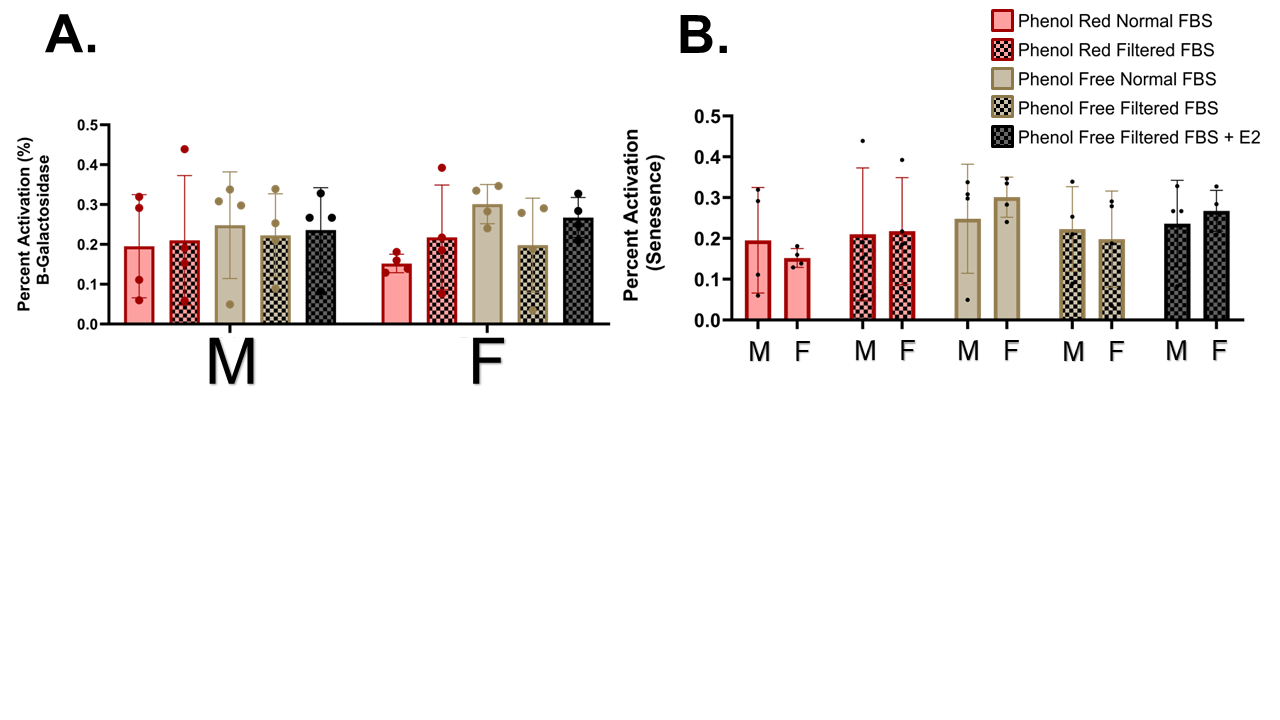
**Figure 17. β-galactosidase-associated senescence staining on Day 5.** Statistical comparisons done within donor sex (A) Statistical comparisons done between donor sexes. (B) Analyzed utilizing 2-way ANOVA and Bonferroni multiple comparison testing. See table for linear model statistics and significance.
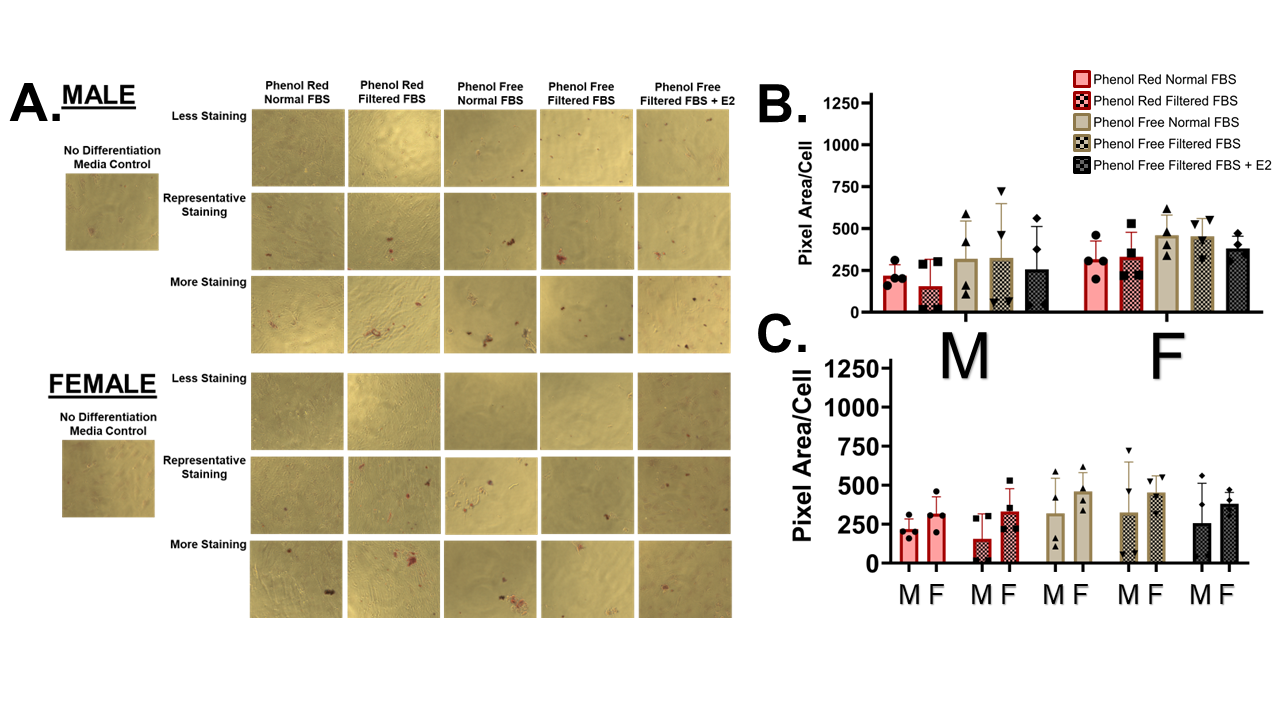
 * P =< .05, ** P =< .01, *** P =< .001, **** P =< .0001. Each dot represents a different donor

**Supplementary Figure 18. Alizarin Red Staining Individual Donors**


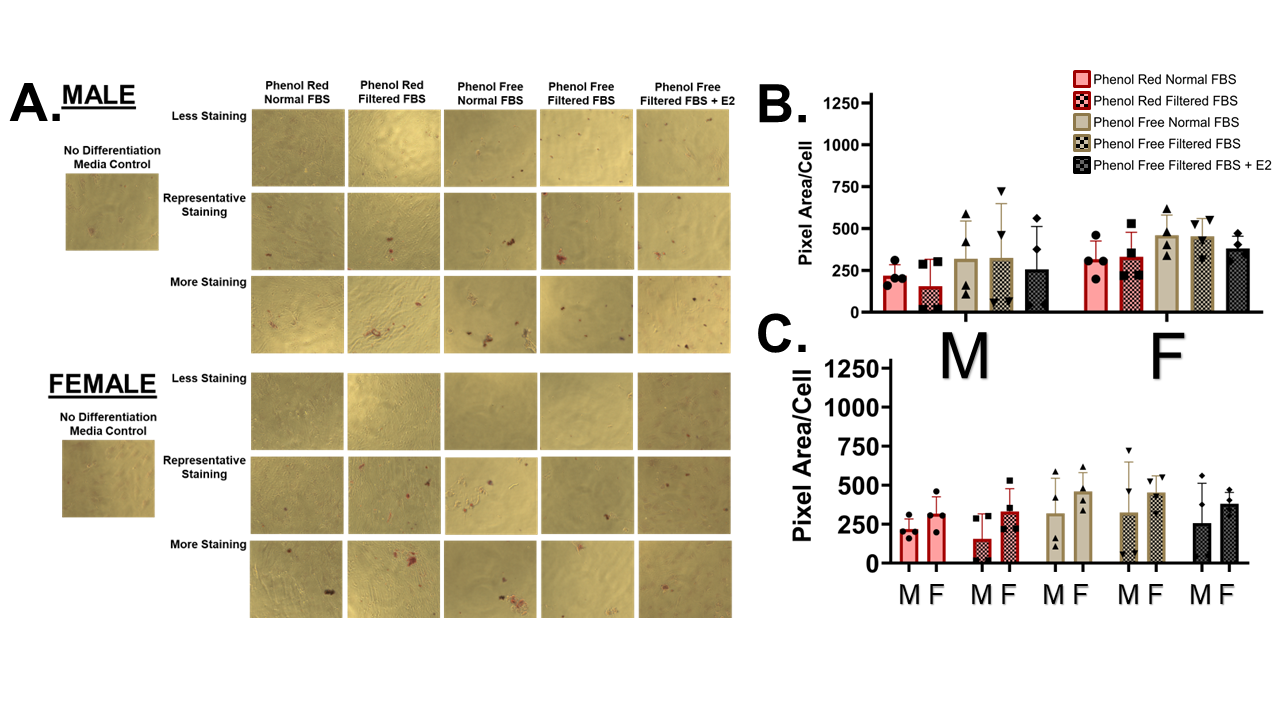
**Figure 18.** **Alizarin Red staining** **to assess media impacts on osteogenic differentiation.** Representative images ranging from less to more staining for each condition in male and female hMSCs. (A) Statistical comparisons done within donor sex. (B) Statistical comparisons done between donor sexes. (C) Analyzed utilizing linear model analysis. For linear model statistics and significance see table later. * P =< .05, ** P =< .01, *** P =< .001, **** P =< .0001. Each point represents a different donor

**Supplementary Figure 19. Oil Red O Differentiation Individual Donors**

**
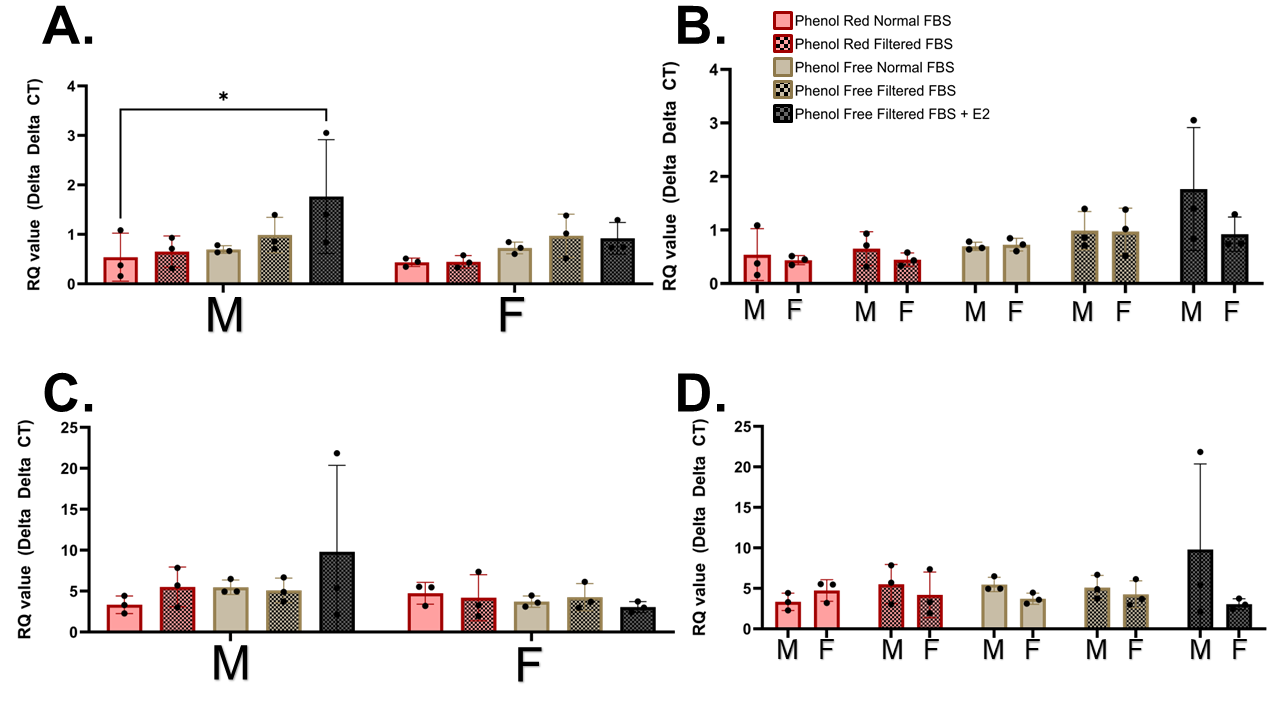
**

**Supplementary Figure 20. Col1a1 and ESR1 Individual Donors**


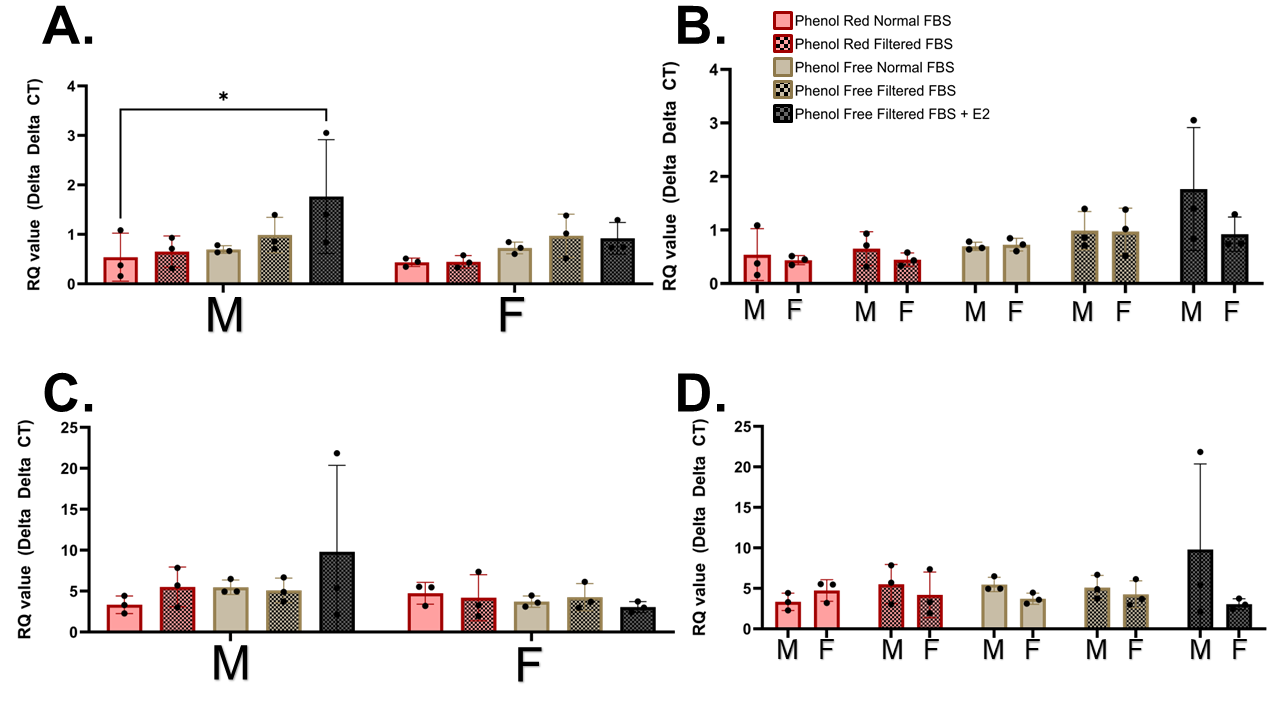
**Figure 20. Col1a1 and ESR1 expression.** Statistical comparisons done within donor sex for Col1a1 (A) and ESR1 (C). Statistical comparisons done between donor sexes for Col1a1 (B) and ESR1 (D). Analyzed utilizing linear model testing. See table for linear model and significance statistics. * P =Z< .05, ** P =< .01, *** P =< .001, **** P =< .0001. Each dot represents a different donor

**Supplementary Figure 21. RUNX2 and Col10a1 Individual Donors**


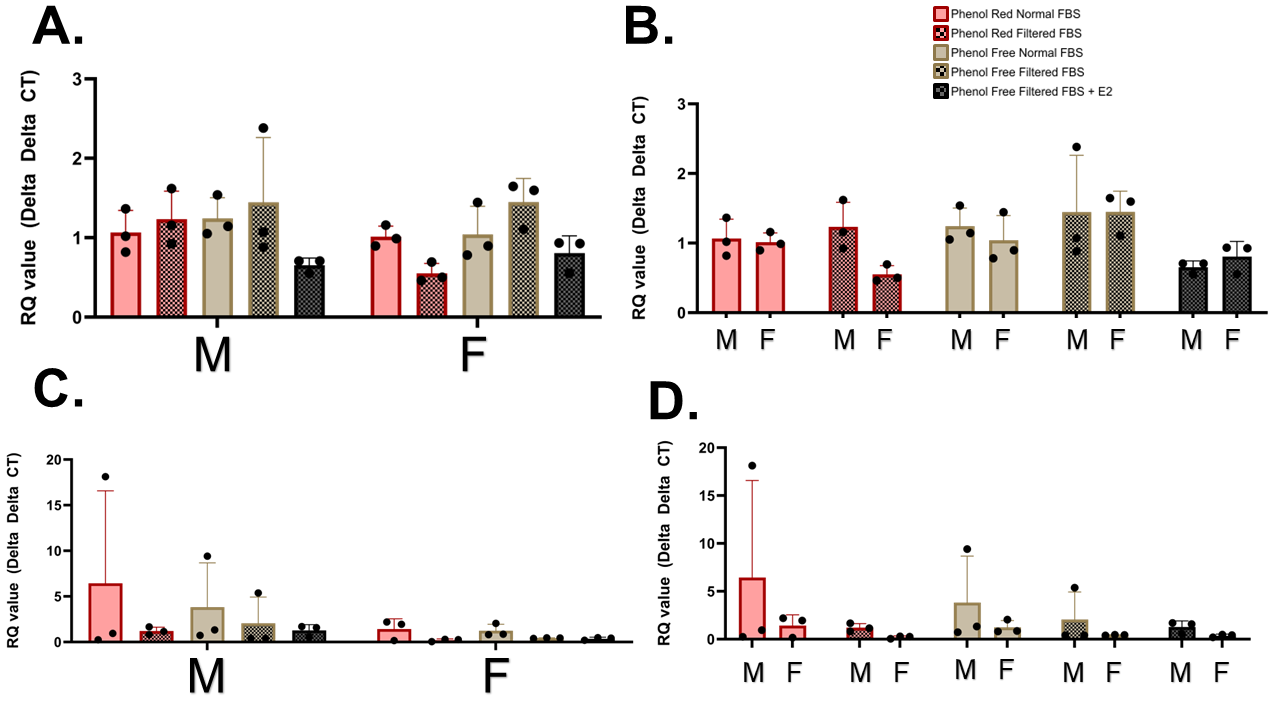
**Figure 9. Osteogenic markers Runx2 and Col10a1.** Statistical comparisons done within donor sex for Runx2 (A) and Col10a1 (C). Statistical comparisons done between donor sexes for Runx2 (B) and Col10a1 (D). For linear model statistics and significance see the table below. * P =< .05, ** P =< .01, *** P =< .001, **** P =< .0001. Each dot represents a different well in a tissue culture plate, 4 wells per donor, 3 donors, for a total of 12 points per condition.

**Supplementary Figure 22. FABP4 and PPARγ Expression Individual Donors**


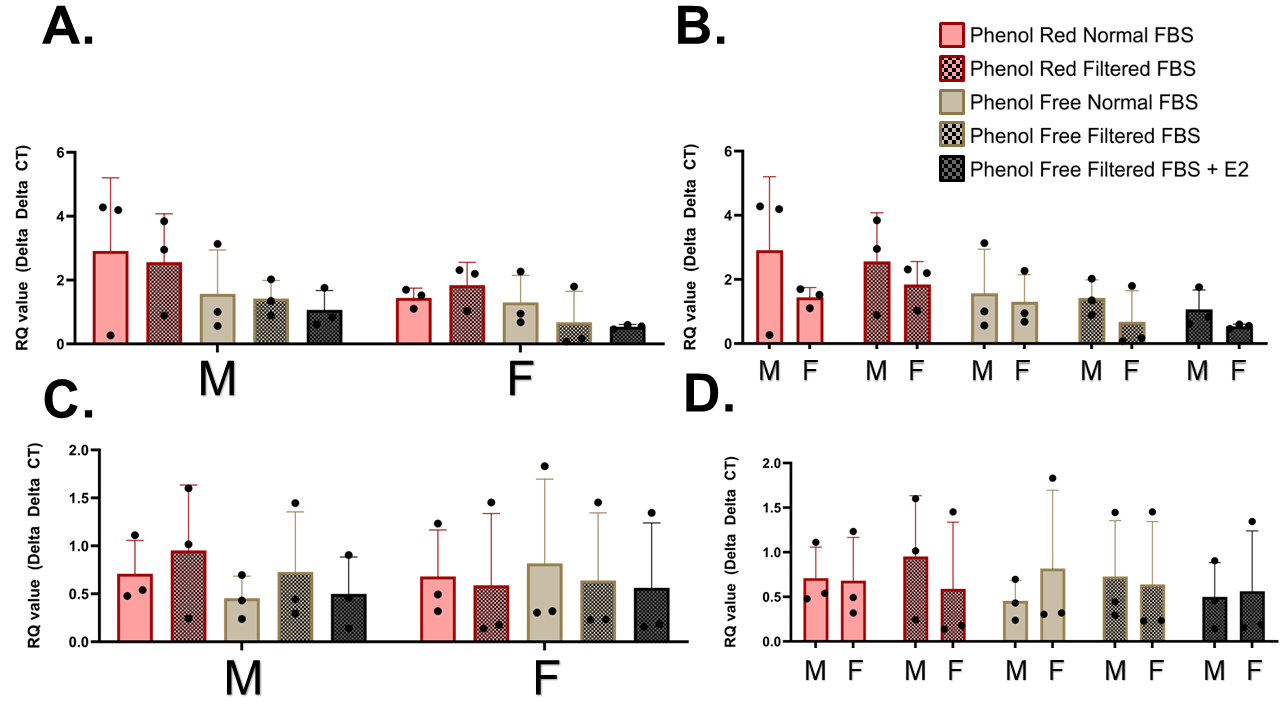
**Figure 22. Adipogenic markers FABP4 and PPARG.** Statistical comparisons done within donor sex for FABP4 (A) and PPARGY (C). Statistical comparisons done between donor sexes for FABP4 (B) and PPARGY (D). For linear model and significance statistics see the table below. * P =< .05, ** P =< .01, *** P =< .001, **** P =< .0001. Each dot represents a different donor

**Supplementary Figure 23. SOX9 Data Individual Donors**


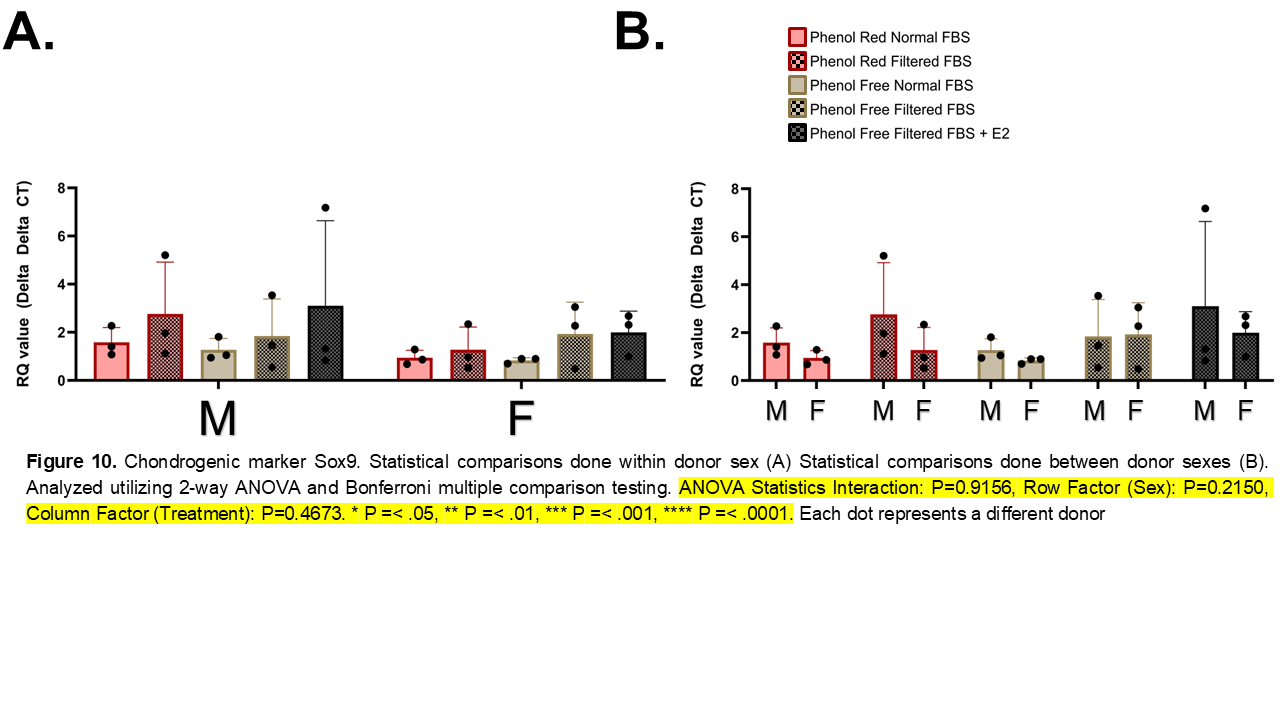


**Figure 11.** **Chondrogenic marker Sox9**. Statistical comparisons done within donor sex (A) Statistical comparisons done between donor sexes (B). Analyzed utilizing linear model analysis. For linear model statistics and significance see the table below. * P =< .05, ** P =< .01, *** P =< .001, **** P =< .0001. Each dot represents a different donor

Supplementary Text 1: Macro Code for IJM using FIJI Senescence

i = 1;

while (i <= 4063) {

//4063

sample = "Sample (" + i + ").jpg";

path = "path to file"+sample;

open(path);

run("Gaussian Blur...", "sigma=5");

run("Colour Deconvolution", "vectors=RGB");

close;

close;

run("Auto Threshold", "method=Triangle");

run("8-bit");

sample_Colour_1 = "Sample (" + i + ").jpg-(Colour_1)";

imageCalculator("Add create", sample, sample_Colour_1);

run("Colour Deconvolution", "vectors=[User values] [r1]=124 [g1]=155 [b1]=111 [r2]=152 [g2]=0.00000 [b2]=255 [r3]=128 [g3]=137 [b3]=255");

close();

run("Gaussian Blur...", "sigma=5");

run("Auto Local Threshold", "method=Phansalkar radius=500 parameter_1=0 parameter_2=0");

run("Analyze Particles...", "size=2000-Infinity circularity=0.20-1.00 show=Overlay display clear summarize");

run("Close All");

i++;

}

Supplementary Text 2: Macro Code for IJM using FIJI Alizarin Red

i = 1;

while (i <= 1650) {

sample = "Sample (" + i + ").jpg";

path = "path to file" + "Sample (" + i + ").jpg";

open(path);

run("Enhance Contrast", "saturated=0.35");

setMinAndMax(0, 255);

run("Gaussian Blur...", "sigma = 2");

run("Colour Deconvolution", "vectors=[User values] [r1]=0.48696724 [g1]=0.57941777 [b1]=0.6535579 [r2]=0.0347683 [g2]=0.5229714 [b2]=0.8516408 [r3]=0.13686699 [g3]=0.39844745 [b3]=0.90692174");

close();

run("Auto Threshold", "method=RenyiEntropy");

run("Invert");

run("Analyze Particles...", " circularity=0.50-1.00 display clear summarize");

run("Close All");

i++;

}

Supplementary Text 3: Macro Code for IJM using FIJI Oil Red O

i = 1;

while (i <= 1650) {

sample = "Sample (" + i + ").jpg";

path = "path to file” + "Sample (" + i + ").jpg";

open(path);

run("Gaussian Blur...", "sigma=5");

run("Colour Deconvolution", "vectors=[User values] [r1]=0.48696724 [g1]=0.57941777 [b1]=0.6535579 [r2]=0.0347683 [g2]=0.5229714 [b2]=0.8516408 [r3]=0.13686699 [g3]=0.39844745 [b3]=0.90692174");

close();

run("Gaussian Blur...", "sigma=5");

run("Auto Threshold", "method=RenyiEntropy");

run("Invert");

run("Analyze Particles...", "size=100-Infinity circularity=0.30-1.00 show=Overlay display clear summarize");

run("Close All");

i++;

}

Table 1: Review of Clinical Trials and their Inclusion of Exogenous Forms of Estrogen


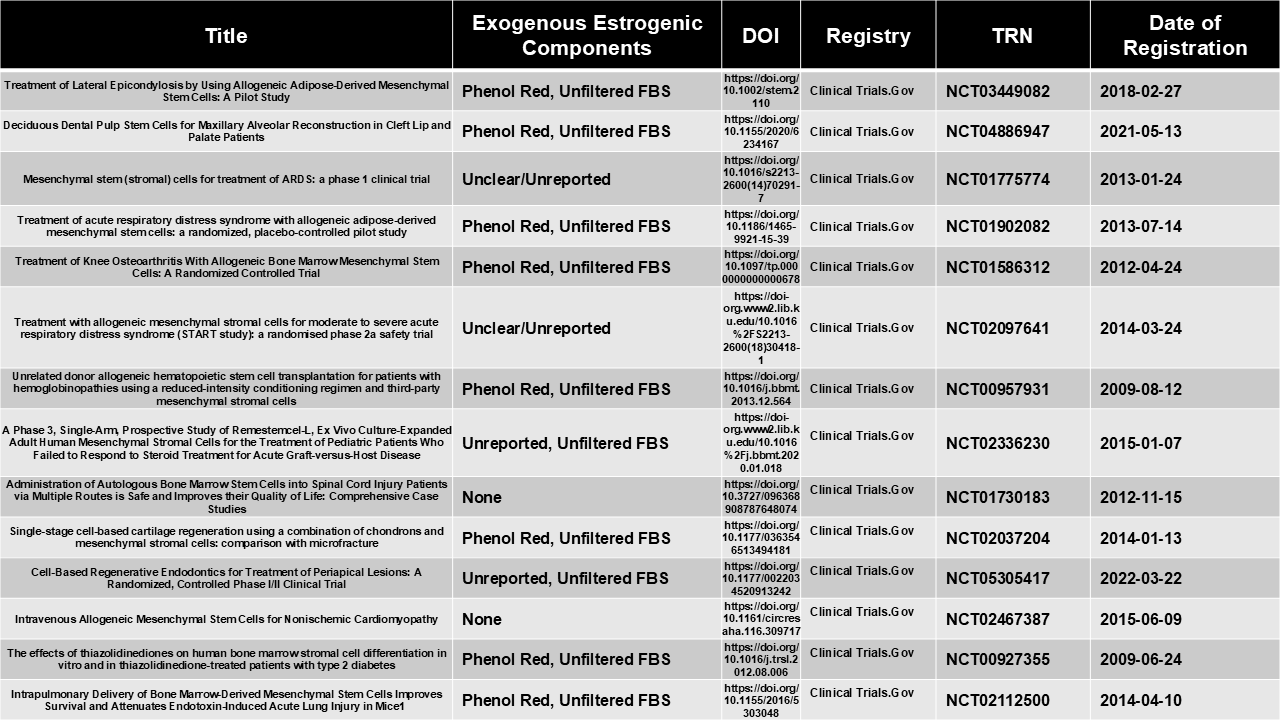


Table 2: Overview of hMSC Donor Sources

| **Donor ID (Shorthand)** | **Donor Characteristics** | **Source** | **Symbol** |
| --- | --- | --- | --- |
| 179 | 21 Male | RoosterBio, Bone Marrow |  |
| 267 | 19 Male | RoosterBio, Bone Marrow |  |
| 334 | 22 Male | RoosterBio, Bone Marrow |  |
| BM-02 | 21 Male | StemBioSys, Bone Marrow |  |
| BM-04 | 21 Female | StemBioSys, Bone Marrow |  |
| 263 | 20 Female | RoosterBio, Bone Marrow |  |
| ATCC | 23 Female | ATCC, Bone Marrow |  |
| 007 | 21 Female | Stemcell Tech, Adipose |  |

Table 3: Linear Modelling of Data with Donors as Data Points


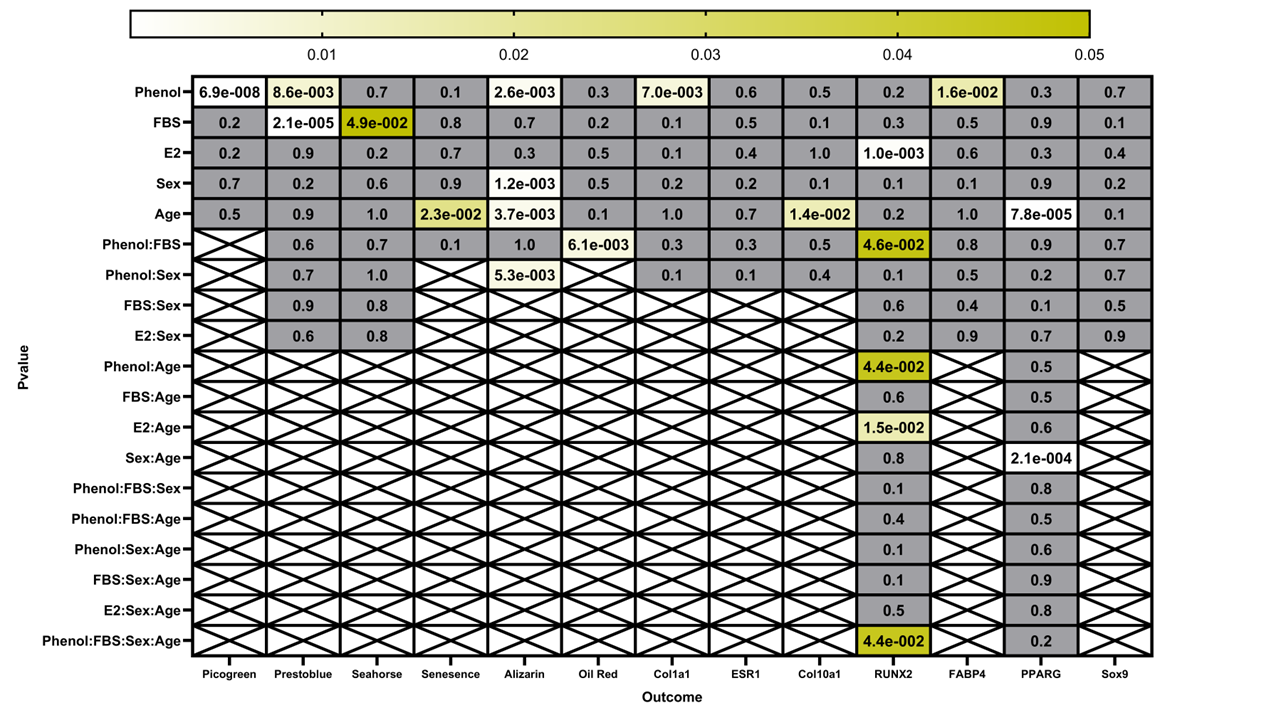

Supplement: Supplementary file 3 — Supplementary Material 3 [file 13293_2026_921_MOESM3_ESM.docx]
